# Impact of an integrated interdisciplinary team-based learning session of medicine and health sector programs at Galala University

**DOI:** 10.1186/s12909-026-09732-4

**Published:** 2026-07-14

**Authors:** Ahmed Nour Eldin Hassan, Noha N Lasheen

**Affiliations:** 1 Faculty of Medicine, 6th October University, Giza, Egypt; 2 Faculty of Medicine, Galala University, Suez, Egypt; 3 Faculty of Medicine, Ain Shams University, Cairo, Egypt

**Keywords:** Interdisciplinary session, Medicine and Health sector, Student-centered Learning, Team-based learning.

## Abstract

**Background and Aim:**

Active learning, especially through interdisciplinary approaches, is increasingly recognized as a means to enhance the effectiveness of health promotion programs. This study evaluated the impact of introducing a team-based learning (TBL) session, structured as an interdisciplinary experience, on students from health-related faculties. The primary aim was to demonstrate the effects of an interdisciplinary TBL session across five faculties at Galala University.

**Methods:**

Using a quasi-experimental pre-post design, an interdisciplinary TBL session was conducted at Galala University, involving students from the Faculties of Medicine, Pharmacy, Physical Therapy, Applied Health Sciences, and Nursing. Twenty-five students volunteered and were divided into five teams. The session, centered on bronchial asthma, followed a three-phase structure: an initial mini-lecture, readiness assurance tests (administered individually and in teams), a debriefing session, and a group application task. At the third phase, students completed a satisfaction survey.

**Results:**

Team-based readiness assurance test (t-RAT) scores consistently exceeded individual readiness assurance test (i-RAT) scores across nine multidisciplinary questions, indicating improved performance through teamwork. The satisfaction survey revealed that the majority of students perceived benefits from participating in the interdisciplinary session.

**Conclusion:**

TBL may play a valuable role in adopting hands-on involvement, peer-assisted learning, critical thinking, and could enrich the student-centered learning through collaborative problem-solving in bronchial asthma.

## Background

Learning is an active process that should involve collaborative efforts between students and instructors to enhance the exchange of knowledge and make it more engaging and enjoyable [1]. One of the most effective and active learning methods is Team-Based Learning (TBL), which emphasizes student-centered learning [2]. In TBL, small groups of students participate in shared activities that apply educational concepts using various skills such as critical thinking, individual and group tasks, and brainstorming. These activities are supported by immediate instructor feedback. TBL was shown to improve students’ communication and teamwork skills, which are essential for delivering high-quality patient care [3]. As a structured instructional approach, TBL promotes active learning through small group teaching. It enables students to collaboratively solve problems, demonstrate decision-making skills, increase motivation, adopt concept mapping, and engage in deep learning [4, 5]. Furthermore, studies have demonstrated that small group discussions with instructors, a core component of TBL, result in better student performance compared to traditional lectures [5].

Conversely, preparation for a Team-Based Learning (TBL) session necessitates that students review pre-recorded lectures and complete mandatory readings before the session, thereby minimizing knowledge gaps within teams. Instructors often use open-ended questions to stimulate more profound discussions and enhance learning outcomes [2]. The TBL process comprises three phases: Phase 1 involves individual preparation of tasks or activities; Phase 2 evaluates both individual and team comprehension of the material through readiness assurance tests, followed by feedback, and Phase 3 encompasses group work tasks [6]. Knowledge is initially assessed individually via the Individual Readiness Assurance Test (i-RAT) and then collectively through the Team Readiness Assurance Test (t-RAT), during which students discuss and agree on answers as a group. A debriefing session follows to clarify any misunderstandings, and students may also participate in a team appeal process, defending their answers with evidence-based reasoning. The group task serves as an evaluation tool, enabling students to apply knowledge in relevant contexts and develop psychomotor skills [7].

Traditionally, higher education curricula have concentrated on a single discipline; however, multidisciplinary, interdisciplinary, and transdisciplinary approaches were shown to enhance affective and cognitive learning, cultivate critical thinking, and broaden students’ general knowledge [8]. Achieving these advantages requires the integration of student-centered and interactive teaching strategies to promote active learning and heightened student engagement [9].

Therefore, it is valuable to distinguish between multidisciplinary, interdisciplinary, and transdisciplinary learning, as these concepts represent a continuum from partial to full integration of disciplines [10]. Multidisciplinary learning involves the parallel analysis of a theme or problem by several disciplines, each offering its perspective without true integration. In contrast, interdisciplinary learning emphasizes the integration and interaction between disciplines, encouraging students to synthesize knowledge from multiple fields and develop new perspectives on the discussed topic. Interdisciplinarity acknowledges the contributions of each discipline and may lead to novel disciplinary insights [11–13]. Meanwhile, transdisciplinary learning involves the synthesis of new knowledge from various disciplines into a unified field, often through the learner’s exploration and integration of fragmented disciplinary knowledge [11]; therefore, it depends on the learner’s exploration of the fragmented disciplines to establish the new synthesis [14].

Recently, interdisciplinary education has been adopted to help students cross disciplinary boundaries and better integrate knowledge from multiple fields, enabling them to explain complex phenomena and solve problems that cannot be addressed by a single discipline alone [15–17]. Furthermore, teamwork activities enhance collaboration and problem-solving abilities [12–15]. Although most interdisciplinary courses are offered at advanced undergraduate or postgraduate levels, it is highly beneficial for undergraduate students to participate in multiple integrative learning sessions to expand their understanding of related disciplines [14].

To the best of the authors’ knowledge, there is limited information regarding the implementation and impacts of interdisciplinary, integrated TBL sessions for undergraduate students in medical and health-related fields in the region. Therefore, this study highlights the evidence of interdisciplinary TBL efficacy in a Middle Eastern context, bridging hospital-university gaps absent in prior Western-centric literature.

## Methods

This study aimed at evaluating the impact of an interdisciplinary team-based learning (TBL) session conducted across five faculties at Galala University.

### Study design

This study employs a quasi-experimental pre-post design lacking randomization or control groups, a frequent approach in educational research for evaluating single interventions. It emphasizes within-subject gains, from individual to team performance, over between-group contrasts.

### Sampling process

**Fig. 1:**
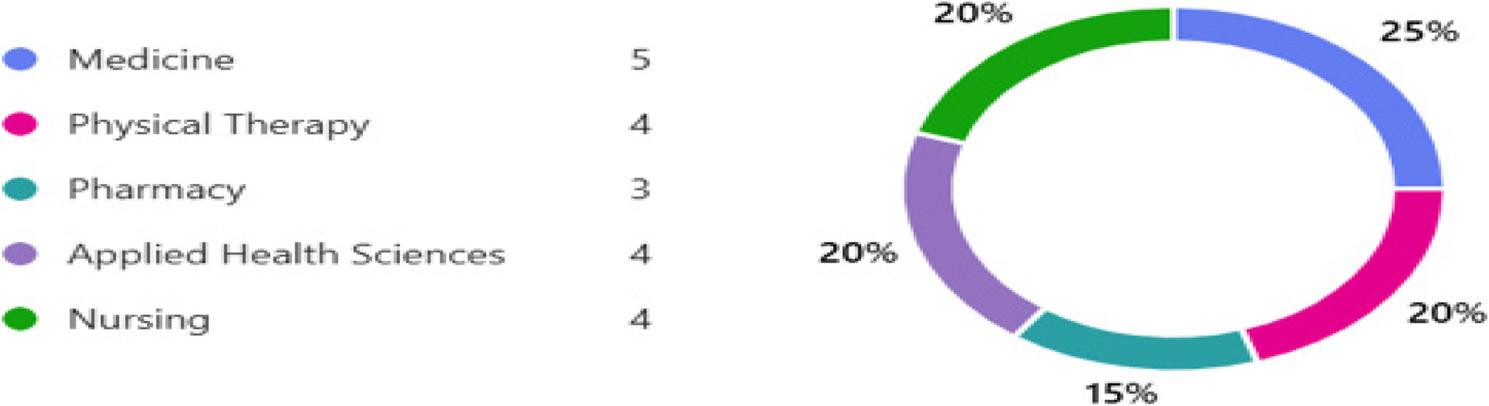
Number and percentage of each GU program in the interdisciplinary TBL session

In this study, the population was undergraduate students from Galala University’s health-related programs (Medicine and Surgery, Pharmacy, Physical Therapy, Nursing, Medical Laboratory Technologies). Strata were defined by program type, with second- and third-year students invited by program directors; volunteers were then randomly assigned to five teams (one or two per program per team) for the interdisciplinary TBL session, as illustrated in Fig. 1, yielding 25 participants with GPAs of 3.0–4.0. This approach ensures each health program is proportionally represented, reducing bias compared to simple random sampling.

### Inclusion criteria

These define eligible participants who align with study goals:

- Enrolled in second- or third-year health sector programs at Galala University.
- Cumulative GPA between 3.0 and 4.0 (indicating academic competence for TBL).
- Voluntary consent, with the right to withdraw without academic penalty.

### Exclusion criteria

These eliminate risks to validity, safety, or ethics:

- First-year or senior students (to focus on mid-level interdisciplinary readiness).
- GPA below 3.0 (potentially indicating a lack of foundational skills for TBL demands).
- Non-volunteers or individuals unable to commit (ensuring ethical participation and engagement).

The selected topic for the session was bronchial asthma. Instructors delivered a mini-lecture addressing the subject from various disciplinary perspectives. Ethical approval for the study was obtained from the Faculty of Medicine’s Ethics Committee at Ain Shams University (FMASU R102).

Intended Learning Outcomes (ILOs) of the Interdisciplinary Session:

- Comprehend the assessment and treatment of patients with severe asthma in the emergency room (covered by Physiology, Health Science, and Clinical Pharmacology).
- Predict potential complications of severe asthmatic attack (covered by Applied Health Sciences).
- Describe potential triggers of asthma attacks (covered by Nursing).
- Identify symptoms that should be reported to the nurse (covered by Nursing).
- Propose management strategies for asthma (covered by Clinical Pharmacology).
- Recognize the role of physiotherapists in asthma treatment (covered by Physical Therapy).
- Describe effective drug formulations used in asthma treatment (covered by Clinical Pharmacy).

This TBL session was designed, moderated, and conducted by Professor Dr. Ahmed Nour El Deen Hassan, Professor of Clinical Pharmacology.

### Study procedure

Students were randomly assigned to five teams, designated as 1 through 5. Prior to the session, they completed integrated lectures prepared by the instructors as pre-class preparation. The session commenced with a 60-minute mini-lecture led by the moderator, which introduced the clinical problem of bronchial asthma and outlined the role of each discipline in its diagnosis and management. Subsequently, students individually completed an Individual Readiness Assurance Test (i-RAT) comprising nine questions, and their mean scores were calculated. The same questions were then answered collaboratively by the teams in the Team Readiness Assurance Test (t-RAT), with team mean scores computed accordingly. To mitigate memorization effects, the order of questions and answer choices was rearranged between the i-RAT and t-RAT. These questions were developed based on the learning objectives and targeted students’ comprehension and analytical skills, aligned with Bloom’s taxonomy.

Each team was then presented with a clinical case accompanied by 10 interdisciplinary questions to be answered within 60 min using any available resources. These questions emphasized the specific contributions of each discipline to the pathophysiology, diagnosis, and management of bronchial asthma, including pharmacological, non-pharmacological, and patient education aspects. Instructors from all participating disciplines reviewed and discussed the answers. At the conclusion of the session, an electronic and anonymous student satisfaction survey was administered. This survey was adopted from the instrument used in the study by Parthasarathy et al. [18].

The nine questions in the i-RAT and t-RAT are mentioned in Table 1. The student satisfaction survey is mentioned in Table 2.

**Table 1:** The Multiple-Choice Questions (MCQs) used in i-RAT and t-RAT

Questions	Choices (Correct answer is in bold)
1) The wheezes in case of bronchial asthma are mainly in which respiratory phase? (1 Point)	A.Allover breathing **B.Expiratory** C.Inspiratory D.Present continuously
2) Which of the following is/are the measure(s) in ABG? (1 Point)	A.Pa O2 B.PaCo2 **C.PaO2**,** PaCo2**,** pH** D.pH
3) Which one is not a routine therapy in treatment of bronchial asthma? (1 Point)	A.Anti-inflammatory **B.Antibiotic** C.Bronchodilators D.Corticosteroids
4) For removal of lung secretion, the patient should be in which of the following position? (1 Point)	**A.According to lung lobe** B.Lying position C.Semi setting position D.Upright position
5) Which of the following is a complication during treatment of exacerbation of bronchial asthma? (1 Point)	A.Cardiac arrest B.Hypokalemia C.Respiratory failure **D.All of the above**
6) Which of the following diameter is suitable for lung deposition with aerosols? (1 Point)	A.5 μm B.< 1 μm C.>5 μm **D.1–5 μm**
7) Pulse oximeter is a device used for which of the following condition? (1 Point)	A.Measure CO2 level B.Measure degree of inflammation C.Measure degree of obstruction **D.Measure oxygen saturation**
8) Which of the following is a rapid method for diagnosis of obstruction in asthma? (1 Point)	A.IgE measure in blood B.Nitric oxide level in breath **C.Peak flow meter** D.Spirometry
9) Which of the following drugs is used as reliever by inhalation route during acute attack? (1 Point)	A.Aminophylline B.Formoterol **C.Formoterol+ Inhaled corticosteroid** D.Inhaled corticosteroid

**Table 2:** The Student Satisfaction Survey Employed in the Present Study

Question	Choices
1) Select Your Program	•Medicine •Physical Therapy •Pharmacy •Applied Health Sciences •Nursing
2) On a Likert scale from 1 = strongly disagree to 5 = strongly agree, the following points were asked:	•I spent time studying before TBL session to be well-prepared •I contributed to my team members’ learning •I enjoyed TBL activities •I learned better in a team setting TBL activities are an effective approach to learning •I am easily distracted during TBL activities •I got bored during TBL activities •I easily remember what I learned when working in a team •TBL activities help me to recall past information •I remember information for longer time when I go over it with team members •I remembered the studying materials easier after applying exercises used in TBL •TBL activities are a waste of time •TBL will help me to improve my scores •The duration of TBL session was appropriate •The venue of TBL session was suitable •This TBL session was well-organized •The time and date of TBL were appropriate •I have had a good experience with TBL •I advise my colleagues to attend TBL sessions •I wish to repeat TBL sessions regularly

### Data analysis

The data analysis was conducted using SPSS version 31 (Chicago, USA). The paired “t” test was employed to compare the i-RAT and t-RAT, as well as their respective mean scores. Additionally, the Pearson correlation coefficient was utilized to assess the validity of the questionnaire, while Cronbach’s Alpha was employed to evaluate its reliability.

Given the modest sample size (*N* = 20), primary analyses focused on effect size estimation and 95% confidence intervals to quantify the magnitude, direction, and precision of effects, supplementing traditional null hypothesis significance testing. This approach mitigates limitations of P-values in low-power settings by prioritizing practical significance over binary decisions at α = 0.05 (two-sided). All analyses were conducted using SPSS version 31, with assumptions (normality) verified via Q-Q plots.

## Results

### Reliability of the questionnaire

To ascertain the reliability of the questionnaire, Cronbach’s Coefficient Alpha was employed as a measure of internal consistency. The normal range for Cronbach’s Coefficient is between 0.0 and + 1.0, with higher values indicating a greater degree of internal consistency. The Cronbach’s Coefficient Alpha was calculated for each item of the questionnaire. The Cronbach’s Alpha for the utilized questionnaire ranged from 0.7 to 0.717, demonstrating the reliability of each item. As depicted in Table 2, the Cronbach’s Alpha for the entire questionnaire is 0.721, indicating a highly reliable questionnaire. This is further corroborated by Tables 3 and 4.

**Table 3:** Reliability test of satisfaction survey

Cronbach’s Alpha	Cronbach’s Alpha Based on Standardized Items	No of Item
0.721	0.877	20

**Table 4:** Reliability test for each item in the satisfaction survey

Satisfaction item	Cronbach’s Alpha if Item Deleted
I spent time studying before TBL session to be well-prepared	0.703
I contributed to my team members’ learning	0.713
I enjoyed TBL activities	0.712
I learned better in a team setting	0.716
TBL activities are an effective approach to learning	0.717
I am easily distracted during TBL activities	0.714
I got bored during TBL activities	0.706
I easily remember what I learned when working in a team	0.711
TBL activities help me to recall past information	0.709
I remember information for longer time when I go over it with team members	0.709
I remembered the studying materials easier after applying exercises used in TBL	0.707
TBL activities are a waste of time	0.716
TBL will help me to improve my scores	0.705
The duration of TBL session was appropriate	0.700
The venue of TBL session was suitable	0.701

### Descriptive values of satisfaction survey items

As depicted in Table 5, statistical analysis of each item in the satisfaction survey revealed that positive indicators, such as enjoyment of TBL activities and enhanced learning in a team environment, exhibited mean values ranging from 3.95 to 4.75. Conversely, negative indicators, including susceptibility to distraction during TBL activities and boredom during TBL activities, demonstrated a range of 2.05 to 2.85. The overall mean satisfaction score was 81.55.

**Table 5:** Mean and standard deviation of each survey item

Survey item	Mean	Std. Deviation
I spent time studying before TBL session to be well-prepared	2.85	1.46
I contributed to my team members’ learning	4.45	1.05
I enjoyed TBL activities	4.80	0.52
I learned better in a team setting	4.75	0.55
TBL activities are an effective approach to learning	4.90	0.31
I am easily distracted during TBL activities	2.10	1.37
I got bored during TBL activities	2.05	1.54
I easily remember what I learned when working in a team	4.65	0.59
TBL activities help me to recall past information	4.75	0.55
I remember information for longer time when I go over it with team members	4.65	0.59
I remembered the studying materials easier after applying exercises used in TBL	4.65	0.67
TBL activities are a waste of time	1.35	0.93
TBL will help me to improve my scores	4.45	0.83
The duration of TBL session was appropriate	4.20	1.11
The venue of TBL session was suitable	4.30	0.98
This TBL session was well-organized	4.55	0.83
The time and date of TBL were appropriate	3.95	1.47
I have had a good experience with TBL	4.70	0.92
I advise my colleagues to attend TBL sessions	4.75	0.55
I wish to repeat TBL sessions regularly	4.70	0.57
Total	**81.55**	**8.33**

### Validity of the satisfaction survey

Pearson’s correlations between all items of the student satisfaction survey revealed consistently strong positive correlations among related items, indicating good internal consistency. For instance, “I enjoyed TBL activities” and “I learned better in a team setting” show a high correlation (0.800, *P* < 0.01), supporting internal consistency. Additionally, divergent (discriminant) validity was analyzed, such as low or negative correlations between unrelated items. For example, “I got bored during TBL activities” is negatively correlated with “I enjoyed TBL activities” (-0.462, *P* < 0.05), which was anticipated. Regarding strong positive correlations, numerous items related to positive TBL experiences (enjoyment, teamwork, effective learning) are strongly and significantly correlated, supporting construct validity and internal consistency. As regards expected negative correlations, items reflecting negative experiences (namely boredom, distraction) are negatively correlated with positive experience items, supporting discriminant validity. Regarding significance levels, many correlations are significant at the 0.01 or 0.05 levels, indicating that these relationships are unlikely to be due to chance. Most items have moderate to high correlations with the total score, indicating that each item contributes to the overall measurement of the construct.

There were significantly positive correlations between the total score and the following survey items:

- Spending more time before TBL to be well-prepared.
- Enjoying TBL activities.
- Effectiveness of TBL activities in learning.
- Easy recall of learned information when working in a team.
- TBL activities aiding in recall of past information.
- Long-term retention of information when working with a team.
- Easier memorization of study materials after applying exercises used in TBL.
- TBL’s ability to improve student scores.
- Appropriate duration of TBL session.
- Suitable venue of TBL session.
- Appropriate time and date of TBL.
- Desire to repeat TBL sessions regularly.

These correlations are presented in Table 6.

**Table 6:** Pearson’s correlations of total score with each survey item

Survey item		Pearson’s correlation
I spent time studying before TBL session to be well-prepared	Pearson Correlation	0.491^*^
	Sig. (2-tailed)	0.028
	N	20
I contributed to my team members’ learning	Pearson Correlation	0.361
	Sig. (2-tailed)	0.118
	N	20
I enjoyed TBL activities	Pearson Correlation	0.558^*^
	Sig. (2-tailed)	0.011
	N	20
I learned better in a team setting	Pearson Correlation	0.353
	Sig. (2-tailed)	0.127
	N	20
TBL activities are an effective approach to learning	Pearson Correlation	0.515^*^
	Sig. (2-tailed)	0.020
	N	20
I am easily distracted during TBL activities	Pearson Correlation	0.322
	Sig. (2-tailed)	0.167
	N	20
I got bored during TBL activities	Pearson Correlation	0.441
	Sig. (2-tailed)	0.051
	N	20
I easily remember what I learned when working in a team	Pearson Correlation	0.558^*^
	Sig. (2-tailed)	0.011
	N	20
TBL activities help me to recall past information	Pearson Correlation	0.652^**^
	Sig. (2-tailed)	0.002
	N	20
I remember information for longer time when I go over it with team members	Pearson Correlation	0.633^**^
	Sig. (2-tailed)	0.003
	N	20
I remembered the studying materials easier after applying exercises used in TBL	Pearson Correlation	0.639^**^
	Sig. (2-tailed)	0.002
	N	20
TBL activities are a waste of time	Pearson Correlation	0.285
	Sig. (2-tailed)	0.223
	N	20
TBL will help me to improve my scores	Pearson Correlation	0.605^**^
	Sig. (2-tailed)	0.005
	N	20
The duration of TBL session was appropriate	Pearson Correlation	0.611^**^
	Sig. (2-tailed)	0.004
	N	20
The venue of TBL session was suitable	Pearson Correlation	0.637^**^
	Sig. (2-tailed)	0.003
	N	20
This TBL session was well-organized	Pearson Correlation	0.397
	Sig. (2-tailed)	0.083
	N	20
The time and date of TBL were appropriate	Pearson Correlation	0.471^*^
	Sig. (2-tailed)	0.036
	N	20
I have had a good experience with TBL	Pearson Correlation	0.351
	Sig. (2-tailed)	0.129
	N	20
I advise my colleagues to attend TBL sessions	Pearson Correlation	0.433
	Sig. (2-tailed)	0.056
	N	20
I wish to repeat TBL sessions regularly	Pearson Correlation	0.589^**^
	Sig. (2-tailed)	0.006
	N	20
Total	Pearson Correlation	1

As *N* = 20, Type I risks are addressed via an effect size focus

*: Correlation is significant at the 0.05 level (2-tailed)

**: Correlation is significant at the 0.01 level (2-tailed)

### Changes in the i-RAT, the t-RAT, and mean score

**Fig. 2:**
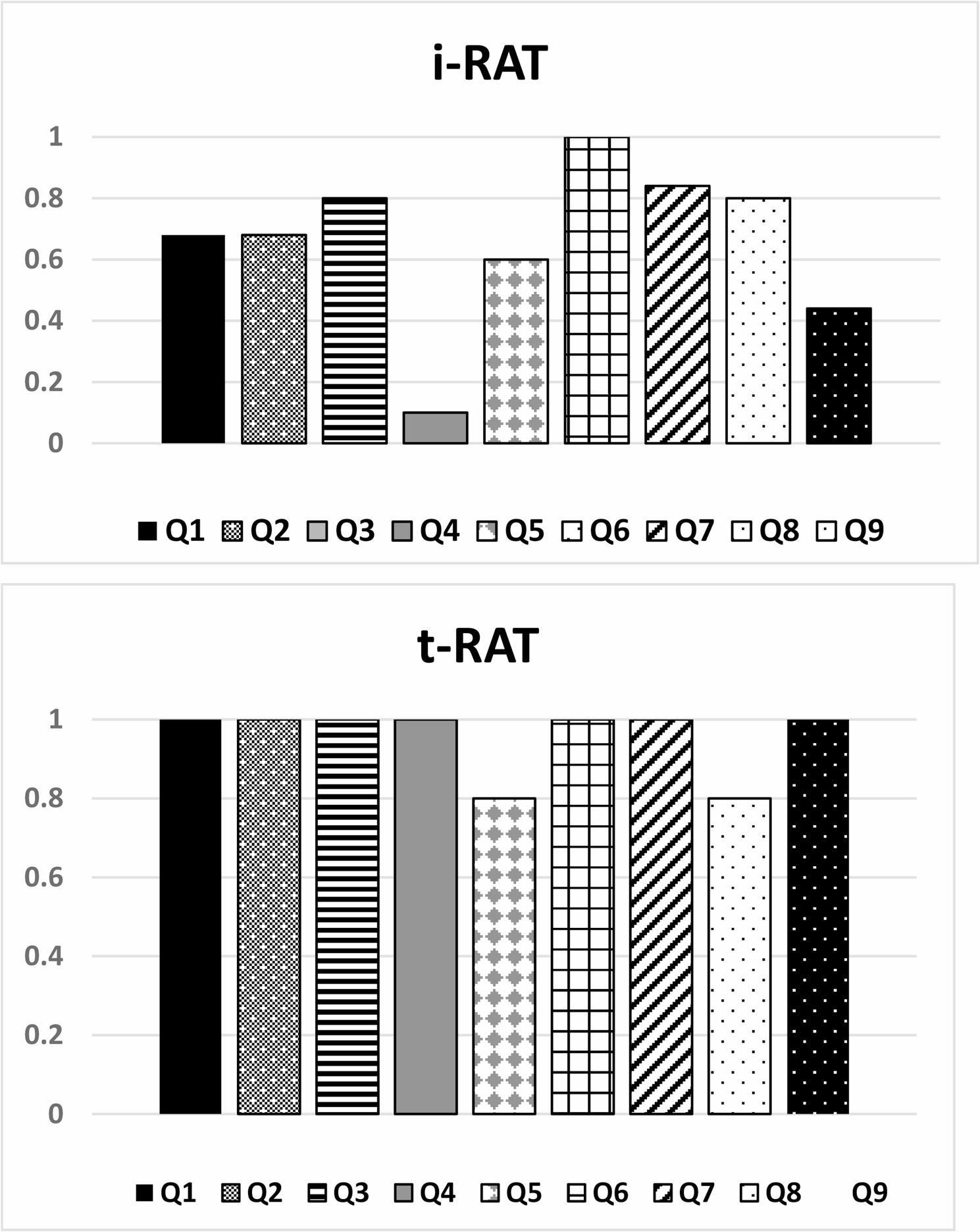
i-RAT and t-RAT of the interdisciplinary TBL session

**Fig. 3:**
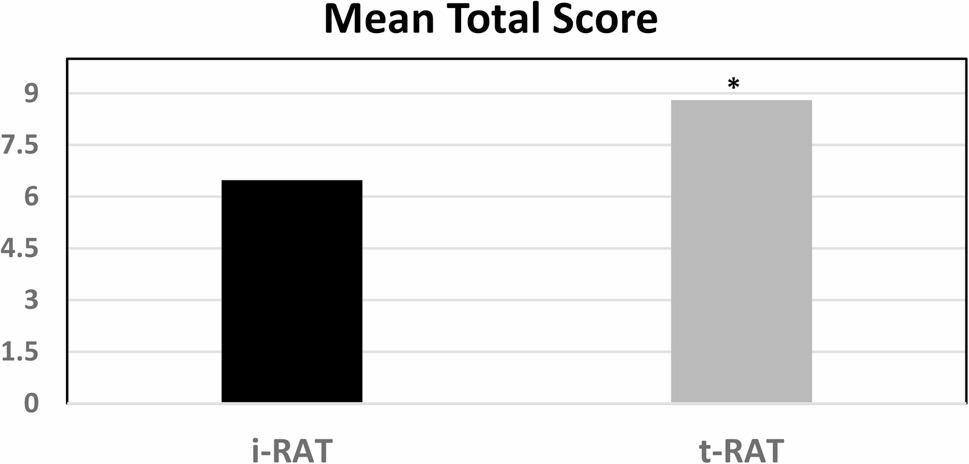
Mean score in i-RAT and t-RAT in the interdisciplinary session

On comparing the Team Readiness Assurance Test (t-RAT) scores with the Individual Readiness Assurance Test (i-RAT) scores, significant improvements were observed across the nine questions (*P* < 0.05). Notably, seven out of the nine questions achieved the maximum score during the t-RAT, as illustrated in Fig. 2. Additionally, the mean score for the t-RAT was approximately 20% significantly higher than that of the i-RAT (*P* < 0.02), as shown in Fig. 3.

Figure 1 depicts the distribution of students from each faculty, indicating that each program contributed between 15% and 25% of the members within each team.

### Responses of students for the student satisfaction survey

Regarding student satisfaction, the majority either strongly agreed or agreed with several positive statements. These included spending adequate time preparing before the TBL session, enjoying the TBL activities, learning more effectively in a team setting, and recognizing TBL as an effective active learning approach. Students also reported that working in teams facilitated easier and longer-lasting recall of information, enhanced the retrieval of previously learnt material, and improved immediate performance following the application exercises used in TBL. Furthermore, they agreed that TBL could improve their academic performance, particularly when sessions were conducted for an appropriate duration and in a suitable venue. They also emphasized that well-organized sessions contributed to a positive overall experience. Many students expressed willingness to recommend TBL sessions to their peers and supported the idea of regular repetition of such sessions.

Conversely, students disagreed with statements suggesting that preparing for the TBL session was time-consuming, that they felt bored during TBL activities, or that they were easily distracted during these sessions.

**Fig. 4:**
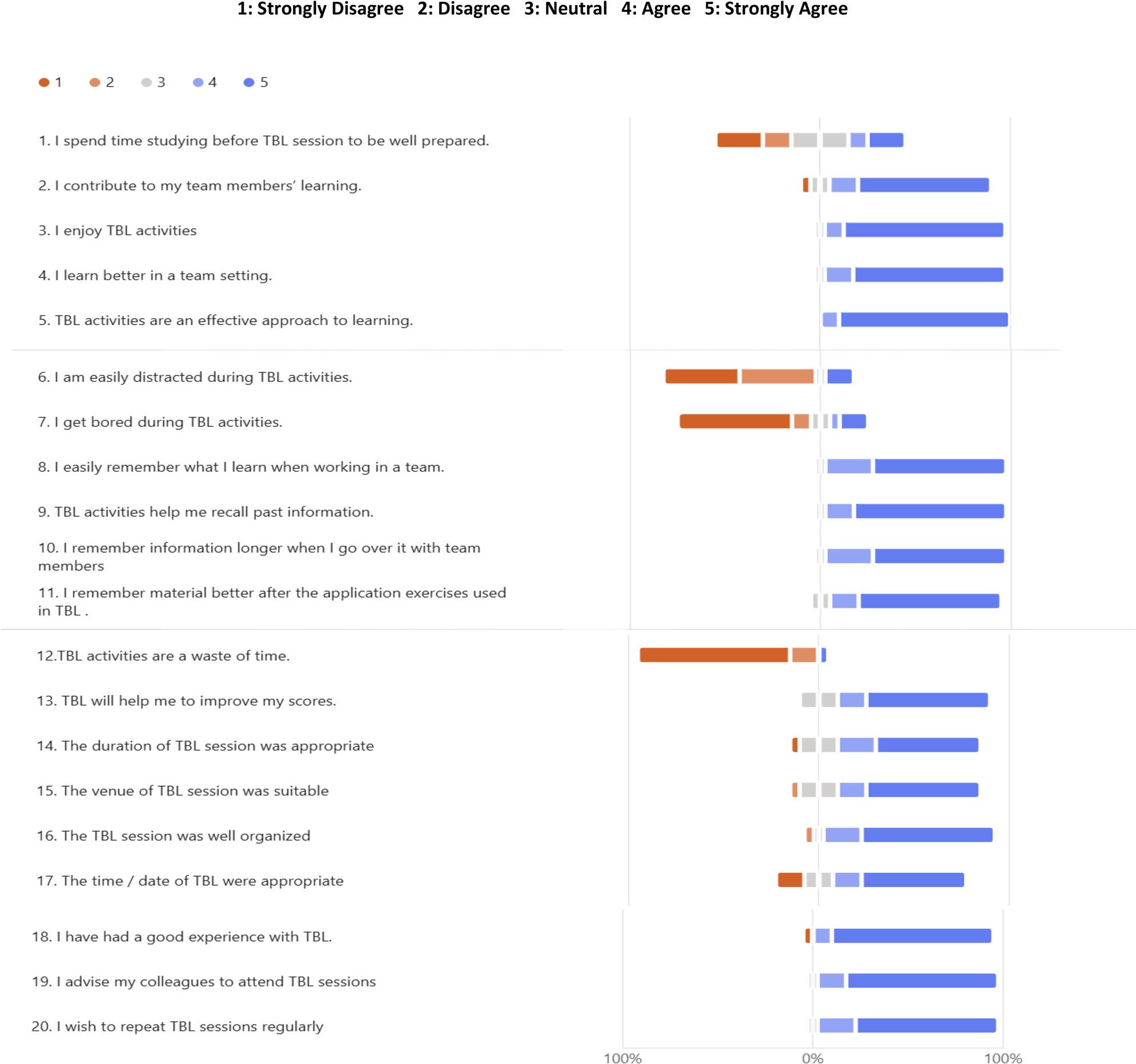
Responses for student satisfaction survey about TBL interdisciplinary session

Furthermore, participants affirmed that TBL activities were not a waste of time. Positive descriptors such as “great experience,” “best outcome,” and “real-life experience” were frequently used to characterize the interdisciplinary TBL session, reflecting a favorable impression, as summarized in Fig. 4.

**Fig. 5:**
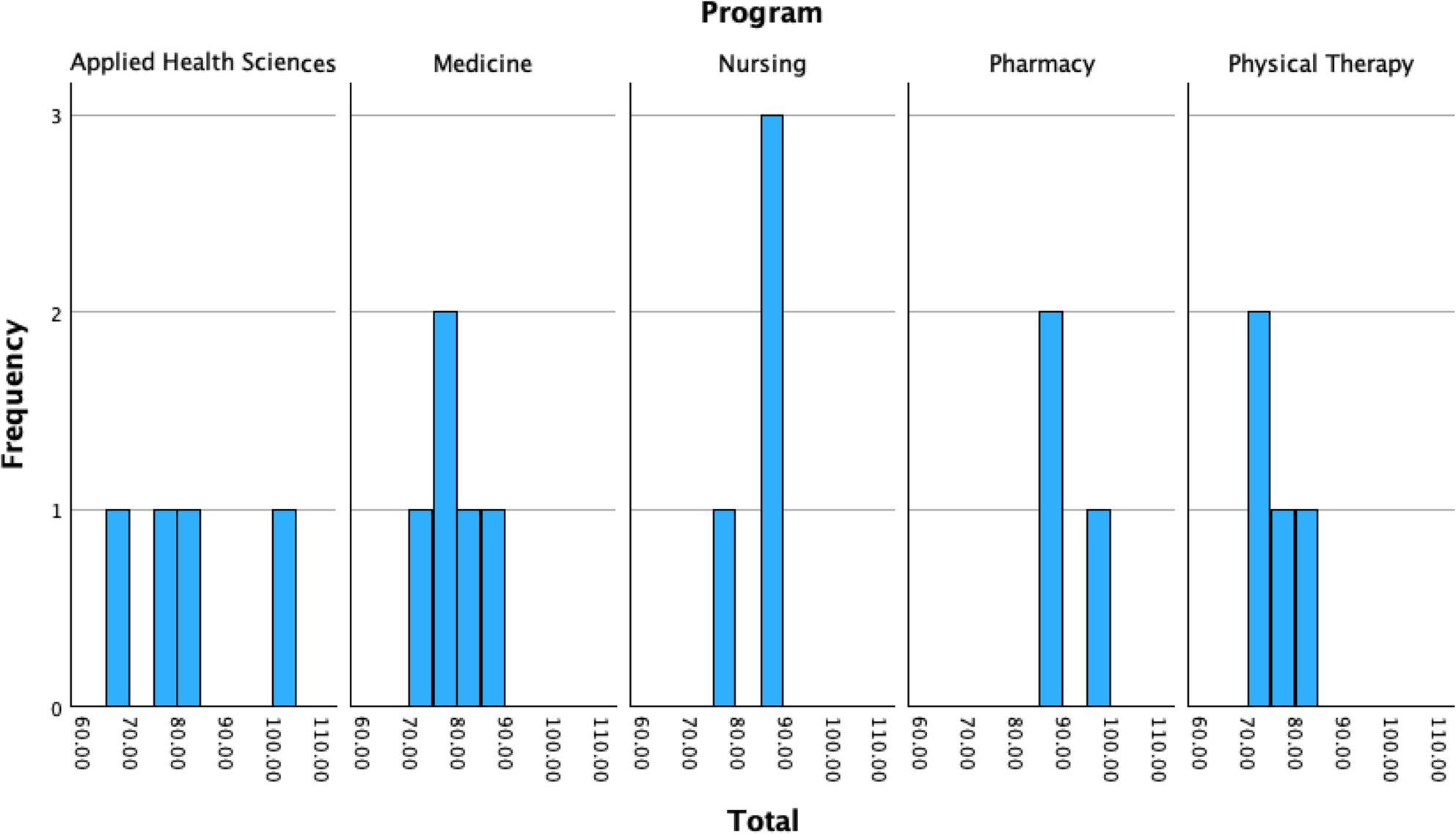
Responses for satisfaction survey divided by GU program

To analyze the responses of students for the satisfaction survey, as shown in Fig. 5, the bar chart displays the distribution of students from five different health-related programs, namely Applied Health Sciences, Medicine, Nursing, Pharmacy, and Physical Therapy, who participated in the interdisciplinary TBL session. The x-axis represents the total number of students in each program, while the y-axis shows the frequency (number of students). Each program is represented by a separate panel. Most programs have either 1 or 2 students in each frequency category. Nursing, pharmacy, and physical therapy have a slightly higher frequency in certain survey score ranges, indicating more satisfaction was expressed by students from these programs who participated in the session.

## Discussion

This study demonstrated for the first time the impact of interdisciplinary TBL sessions in the medical curriculum in the recently established university, Galala university. It revealed that such sessions have the potential to enhance knowledge assimilation and promote effective teamwork, peer-assisted learning, active learning and more student engagement.

This study aligns with the consensus that interprofessional collaborative practice, where multidisciplinary health professionals collaborate as a team, is fundamental to providing safe, high-quality, and effective patient-centred care [19].

The incorporation of real clinical cases into the learning process emphasizes the significance of interdisciplinarity for students’ future professional roles. Collaboration with professionals from diverse backgrounds is essential for optimal health outcomes. Therefore, this study is consistent with previous research that highlights the crucial role of interdisciplinary competencies in academic development and their increasing value in professional practice [20, 21].

In this study, the higher scores on the t-RAT, which may be attributed to the collaborative sharing of information, in addition to the positive survey responses, corroborate Silberman et al.’s findings [22]. These findings demonstrated a positive correlation between interdisciplinary learning, enhanced student participation, cooperative learning, and the development of critical thinking skills. Collaborative question-answering allows students to revisit ideas, defend their choices, and re-evaluate options using higher-order thinking while benefiting from immediate peer feedback. This collaborative process enables students to identify incorrect answers and try again, thereby fostering deeper learning.

This study also highlighted the effectiveness of teamwork within the TBL framework. In support of this, Berasategi et al. [23] observed that interdisciplinary teams benefit from the responsibility to share disciplinary knowledge, maintain a broad overview of the topic, and propose appropriate interventions. Such collaboration motivates team members to achieve a more holistic understanding and collectively resolve challenges. The establishment of a learning community, as illustrated in Fig. 4, was reflected in the students’ experiences during the case study and survey. Collaborating with diverse perspectives may present both challenges and opportunities for personal growth, with teamwork influenced by group diversity and a sense of belonging. During phase 2, students showed initiative in seeking information, creativity, and freedom in problem-solving, as observed by instructors.

It is noteworthy that instructors anticipated some students may feel uncomfortable when participating in team-based and stress management activities. To address this concern, the use of open-ended questions in phase 3 was recommended to help students navigate the diffuse boundaries of the TBL session. These challenges may be partially attributed to the novelty of TBL sessions at Galala university.

Interdisciplinary learning has been shown to facilitate the development of critical thinking and metacognitive skills through discussion, enabling students to appreciate diverse perspectives and integrate knowledge from various disciplines. This approach promotes a more comprehensive understanding of interconnected concepts and encourages students to adopt an interdisciplinary outlook that values the contributions of other fields [20]. Furthermore, interdisciplinarity enhances students’ sense of relatedness to peers from different academic backgrounds.

In line, this study demonstrated that interdisciplinary TBL sessions support teamwork, encourage the exploration of ideas, and foster the development of ethical, social, and professional perspectives. Additional benefits observed included increased enthusiasm for learning, improved problem-solving skills, greater research and inquiry capacity, effective information use, independent work planning, and enhanced analytical abilities [24]. These impacts were evident during group discussions and case analysis in phase 3 of the TBL session.

### Limitations

The study’s statistical power and generalizability to broader populations are limited as it was conducted on 25 students from a single institution in Egypt. The small sample (*N* = 20) constrains statistical power, widening confidence intervals and elevating the risk of Type II errors, though Type I error remains nominally controlled at α = 0.05. Effect sizes were thus emphasized to provide context for observed associations, avoiding overreliance on P-values which can be unstable in small samples.

nother limitation is the lack of long-term assessments of knowledge retention and skill transfer into clinical practice, as the outcomes were measured immediately post-session using short-term i-RAT/t-RAT scores and a self-reported satisfaction survey. Our study focuses only on one topic, which is bronchial asthma, without a control group, further limiting the interdisciplinary benefits of TBL.

### Recommendations

In light of the demonstrated increase in t-RAT scores (a mean increase of approximately 20%, *P* < 0.02), high student satisfaction (a total mean of 81.55/100), and the robust survey reliability (Cronbach’s α = 0.721), it is valuable to increase the integration of interdisciplinary TBL sessions into undergraduate curricula across health-related programs.

Future studies should focus on scaling participant numbers to enhance generalizability, incorporate longitudinal assessments of knowledge retention and clinical application; for example, 6–12-month follow-ups with OSCEs or pre/post clinical performance, across multiple topics and institutions to enhance the external validity. Furthermore, faculty development workshops are fully recommended to standardize TBL facilitation, with an emphasis on diverse team composition and case integration.

## Conclusion

TBL may have a valuable role in adopting hands-on involvement, peer-assisted learning, critical thinking, and active learning through collaborative problem-solving on clinical topics.

## Data Availability

Upon request from the corresponding author, the data will be available.

## Abbreviations

TBL: Team Based Learning
t-RAT: Team-based readiness assurance test
i-RAT: Individual readiness assurance test

## References

[1] Sudarso S, Rahayu GR, Suhoyo Y. How does feedback in mini-CEX affect students’ learning response? Int J Med Educ. 2016;7:407–13. 28008136 10.5116/ijme.580b.363d PMC5198814

[2] Burgess A, Bleasel J, Haq I, Roberts C, Garsia R, Robertson T, Mellis C. Team-based learning (TBL) in the medical curriculum: better than PBL? BMC Med Educ. 2017;17:1–1. 29221459 10.1186/s12909-017-1068-z PMC5723088

[3] O’Daniel M, Rosenstein AH. Chap. 33 Professional Communication and Team Collaboration. In: Hughes RG, editor. Patient Safety and Quality. An Evidence-Based Handbook for Nurses. 2008:3.

[4] Espey M. Enhancing critical thinking using team- based learning. High Educ Res Dev. 2018;37(1):15–29.

[5] Arja SB, Ponnusamy K, Kottathveetil P, et al. Effectiveness of Small Group Discussions for Teaching Specific Pharmacology Concepts. Med Sci Educ. 2020;30:713–8. 34457729 10.1007/s40670-020-00938-9 PMC8368645

[6] Michaelsen LK, Sweet M. Team-based learning. New Dir Teach Learn. 2011;2011(128):41–51.

[7] Zeng R, Xiang L, Zeng J, Zuo C. Applying team- based learning of diagnostics for undergraduate students: assessing teaching effectiveness by a randomized controlled trial study. Adv Med Educ Pract. 2017;8:211. 28331383 10.2147/AMEP.S127626 PMC5352152

[8] Hardy JG, Sdepanian S, Stowell AF, Aljohani AD, Allen MJ, Anwar A, Barton D, Baum JV, Bird D, Blaney A, Brewster L. Potential for chemistry in multidisciplinary, interdisciplinary, and transdisciplinary teaching activities in higher education. J Chem Educ. 2021;98(4):1124–45.

[9] Jariyapong P, Punsawad C, Bunratsami S, Kongthong P. Body painting to promote self-active Mal. J Med Health Sci. 2021;17(2):18–27. 10.3402/meo.v21.30833 PMC4779329 26945229

[10] Klein JT. A taxonomy of interdisciplinarity. In: Frodeman R, Klein JT, Mitcham C, editors. The Oxford Handbook of Interdisciplinarity. Oxford: Oxford University Press; 2012. pp. 15–30.

[11] Petrie HG. Interdisciplinary education: Are we faced with insurmountable opportunities? Rev Res Educ. 1992;18:299–333.

[12] Ivanitskaya L, Clark D, Montgomery G, Primeau R. Interdisciplinary learning: Process and outcomes. Innov High Educ. 2002;27:95–111.

[13] Domik G, Fischer G. Coping with complex real-world problems: Strategies for developing the competency of transdisciplinary collaboration. IFIP Adv Inform Communication Technol. 2010;324:90–101.

[14] Stentoft D. From saying to doing interdisciplinary learning: Is problem-based learning the answer? Act Learn High Educ. 2017;18(1):51–61.

[15] Mansilla Boix. Interdisciplinary work at the frontier: An empirical examination of expert interdisciplinary epistemologies. Issues Integr Stud. 2006;31(24):1–31.

[16] Boix Mansilla and Duraisingh. Targeted Assessment of Students’ Interdisciplinary Work: An Empirically Grounded Framework Proposed. J High Educ. 2007;78(2):215–37.

[17] Spelt EJH, Biemans HJa, Tobi H, Luning Pa, Mulder M. Teaching and learning in interdisciplinary higher education: A systematic review. Educational Psychol Rev. 2009;21:365–78.

[18] Parthasarathy P, Apampa B, Manfrin A. Perception of team-based learning using the team-based learning student assessment instrument: an exploratory analysis within pharmacy and biomedical students in the United Kingdom. J Educ Eval Health Prof. 2019;16:23. 31430842 10.3352/jeehp.2019.16.23 PMC6746672

[19] Panel IECE. Core competencies for interprofessional collaborative practice: Report of an expert panel. Interprofessional Education Collaborative Expert Panel; 2011.

[20] Little A, Hoel A. Interdisciplinary team teaching: An effective method to transform student attitudes. J Eff Teach. 2011;11:36–44.

[21] Carpenter DM, Crawford L, Walden R. Testing the efficacy of team teaching. Learn Environ Res. 2007;10:53–65.

[22] Silberman D, Carpenter R, Takemoto JK, Coyne L. The impact of team-based learning on the critical thinking skills of pharmacy students. Currents Pharm Teach Learn. 2021;13(2):116–21. 10.1016/j.cptl.2020.09.008 33454066

[23] Berasategi N, Aróstegui I, Jaureguizar J, Aizpurua A, Guerra N, Arribillaga-Iriarte A. Interdisciplinary learning at University: Assessment of an interdisciplinary experience based on the case study methodology. Sustainability. 2020;12(18):7732.

[24] Corbacho AM, Minini L, Pereyra M, González-Fernández AE, Echániz R, Repetto L, Cruz P, Fernández-Damonte V, Lorieto A, Basile M. Interdisciplinary higher education with a focus on academic motivation and teamwork diversity. Int J Educational Res Open. 2021;2:100062.

